# Quantitative Analysis of Liver Disease Using MRI-Based Radiomic Features of the Liver and Spleen

**DOI:** 10.3390/jimaging8100277

**Published:** 2022-10-09

**Authors:** Jordan Sack, Jennifer Nitsch, Hans Meine, Ron Kikinis, Michael Halle, Anna Rutherford

**Affiliations:** 1 Division of Gastroenterology, Hepatology, and Endoscopy, Brigham and Women’s Hospital, Boston, MA 02115, USA; 2 Harvard Medical School, Boston, MA 02115, USA; 3 Medical Imaging Computing Group, University of Bremen, D-28359 Bremen, Germany; 4 Surgical Planning Laboratory, Brigham and Women’s Hospital, Boston, MA 02115, USA; 5 Fraunhofer Institute for Digital Medicine, D-28359 Bremen, Germany

**Keywords:** cirrhosis, spleen, diagnosis, severity, radiomics, MRI

## Abstract

Background: Radiomics extracts quantitative image features to identify biomarkers for characterizing disease. Our aim was to characterize the ability of radiomic features extracted from magnetic resonance (MR) imaging of the liver and spleen to detect cirrhosis by comparing features from patients with cirrhosis to those without cirrhosis. Methods: This retrospective study compared MR-derived radiomic features between patients with cirrhosis undergoing hepatocellular carcinoma screening and patients without cirrhosis undergoing intraductal papillary mucinous neoplasm surveillance between 2015 and 2018 using the same imaging protocol. Secondary analyses stratified the cirrhosis cohort by liver disease severity using clinical compensation/decompensation and Model for End-Stage Liver Disease (MELD). Results: Of 167 patients, 90 had cirrhosis with 68.9% compensated and median MELD 8. Combined liver and spleen radiomic features generated an AUC 0.94 for detecting cirrhosis, with shape and texture components contributing more than size. Discrimination of cirrhosis remained high after stratification by liver disease severity. Conclusions: MR-based liver and spleen radiomic features had high accuracy in identifying cirrhosis, after stratification by clinical compensation/decompensation and MELD. Shape and texture features performed better than size features. These findings will inform radiomic-based applications for cirrhosis diagnosis and severity.

## 1. Introduction

Radiographic imaging provides a direct yet static view of the progressive physiological changes inherent in cirrhosis. Radiomics is a method for extracting quantitative features from radiographic images to identify potential novel biomarkers for characterizing and prognosticating disease. Our aim in this paper is to explore whether novel image-based techniques using radiomics may provide an objective method for detecting cirrhosis using common abdominal imaging studies and how these methods are affected by liver disease severity. 

Although general radiographic modalities are widely available and used to assess abdominal pathology, their ability to accurately detect cirrhosis is limited. Ultrasonography has a sensitivity of 52–69% and specificity of 74–89% for assessing cirrhosis. while computed tomography (CT) and magnetic resonance imaging (MR) have a sensitivity of 77–84% and specificity of 53–68% [1,2]. These imaging studies are often reported using subjective qualitative descriptions with few quantitative measurements Furthermore, the reliability of these studies can be influenced by liver disease severity which is clinically classified as compensated or decompensated (ascites, variceal bleeding, or hepatic encephalopathy) and by Model for End-Stage Liver Disease (MELD) score, (an established estimate of mortality based on a composite of serum laboratory values widely used for transplant prioritization) [3]. Imaging may not recognize earlier stages of cirrhosis (called compensated cirrhosis). The MELD score can be affected by fluctuations in laboratory values and may significantly under- or over-estimate liver disease severity for some patients [4]. While there have been efforts to improve the yield of MELD using clinical and serologic information [5], MELD and its sub-components are inherently indirect biochemical measures of the physiological processes in cirrhosis that cannot be quantified radiographically and do not incorporate quantitative radiographic markers. Recently, elastography has become an emerging tool with high sensitivity and specificity for assessing hepatic fibrosis and provides quantitative values that can prognosticate liver disease severity [6,7]; yet its access and use is often limited to clinic settings in which patients have been referred for outpatient liver disease evaluation. Therefore, incorporation of quantitative imaging biomarkers into commonly obtained imaging studies could potentially improve recognition of cirrhosis and liver disease in general. Besides elastography, a variety of imaging approaches have been used to assess liver function, including in MR imaging [8].

Radiomics offers a way to bridge the direct radiographic assessment of the liver with the need for quantitative metrics of liver disease and its progression. Radiomic features are calculated by performing image processing operations on regions of radiological images to capture a variety of measures of organ size, shape, and texture. These metrics are then statistically correlated with existing measures of liver disease, such as MELD score and decompensation status, to identify a subset of salient features. This training process yields a computational model that can then be used to assess additional subjects. Unlike human observations of liver disease such as nodularity of the liver or splenomegaly, radiomic analysis has no a priori model of how liver disease is manifested. Rather, that model is created through the training process. Such a model could be used to provide objective assessment of liver disease severity without additional subjective human interpretation of individual patient imaging.

Radiomics has been used to provide diagnostic and prognostic information for a variety of medical diseases and has recently been used in hepatology. Radiomic analyses in liver diseases [9,10] have primarily focused on detecting hepatocellular carcinoma, including its diagnosis in cases of indeterminate hepatic nodules [11] and for assessing risk of recurrence [12]. A smaller number of studies have explored using radiomics to detect specific manifestations of liver disease such as clinically significant portal hypertension [13] and in staging hepatic fibrosis [14,15,16].

Our goal is to expand radiomic application in detecting cirrhosis by using radiomic-quantified imaging of the liver and spleen. These two organs were chosen as they manifest findings of cirrhosis and portal hypertension that can be easily segmented radiographically. We recently used this approach to test the ability of MR radiomic features against different measures of cirrhosis severity [17]. We now first aim to demonstrate the ability of these MR radiomic features of the liver and spleen to detect the presence of cirrhosis when comparing MR imaging in patients with cirrhosis to those without cirrhosis. Second, we aim to determine how radiomics performs when cirrhosis is further stratified by liver disease severity as characterized by clinical compensation/decompensation or MELD score. Third, we aim to show the relative importance of various radiomics feature classes in the liver and the spleen for assessing different stages of cirrhosis.

## 2. Materials and Methods

### 2.1. Patient Selection

This is a single-center retrospective study of MR images acquired from 1 June 2015 to 1 June 2018 from patients with cirrhosis undergoing hepatocellular carcinoma screening and from patients without cirrhosis undergoing intraductal papillary mucinous neoplasm (IPMN) surveillance. Patient imaging studies were queried using the Mass General Brigham Research Patient Data Registry (RPDR), a research tool for extracting clinical data within the Mass General Brigham healthcare system [18].

The cirrhosis cohort was initially identified using ICD-10 code of cirrhosis (K74) and abdominal MR. Patients were included in this cohort if the diagnosis of cirrhosis was confirmed based on chart review of clinical history along with any one of the following: liver biopsy, elastography, or any liver related decompensation (defined as presence of any variceal hemorrhage, ascites, or hepatic encephalopathy), and the patient had a MR scan performed on a 3 Tesla Siemens Verio scanner using a multiparametric, fat suppressed T1-weighted protocol with gadolinium-based contrast agent and a 5 min post contrast scan. The non-cirrhosis IPMN cohort was initially generated using ICD-10 code of pancreatic cysts (K86.2) with no ICD-10 code of cirrhosis (K74) and abdominal MR. Patients were included if there were no clinical, laboratory, radiographic, or elastography evidence of cirrhosis on chart review, and the patient had a MR scan conducted on the same scanner type, protocol, and contrast agent as the cirrhosis cohort. In both cohorts, if patients had multiple eligible imaging scans, the most recent one was used for analysis. Exclusion criteria were the inability to confirm the presence or absence of cirrhosis on chart review, missing MELD labs, presence or history of hepatocellular carcinoma, hepatic cysts larger than 10 mm, hepatic resection, hepatic ablation, or splenectomy. Institutional Review Board approval was obtained. 

### 2.2. Study Outcomes

The primary outcome of this study was the discrimination ability and characterization of radiomic features of the liver and spleen in detecting cirrhosis when MR images of patients with cirrhosis were compared to those without cirrhosis. Secondary analyses on radiomic discrimination of cirrhosis with varying liver disease severity were performed by stratifying the cirrhosis cohort by either clinical compensation/decompensation or by MELD score (stratified at the median MELD). 

### 2.3. Patient Characteristics

Demographic data on age, sex, and ethnicity were obtained for all patients. Among those with cirrhosis, information was abstracted on liver disease etiology and the presence of liver related decompensation at the time of MR imaging. MELD scores were generated using international normalized ratio, serum sodium, serum bilirubin, and serum creatinine laboratory values that were obtained closest to the MR imaging scan.

### 2.4. Extraction of Radiomic Features and Validation Process

**Figure 1:**
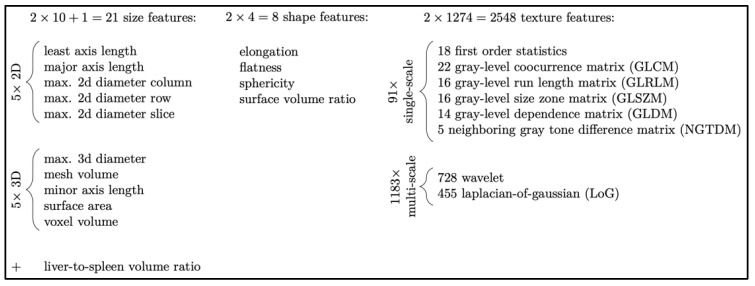
Radiomic features from liver and spleen were divided into high level classes (size, shape, and texture) to better explain their relative importance in each experiment. Note that in other literature, radiomics features of size and shape are typically combined into a single class. Distinct size and shape classes better correspond to human observations such as gross organ size and texture features such as surface nodularity, respectively.

Feature analysis was performed on the contrast-enhanced imaging acquisition [17]. Due to the homogenous imaging conditions described above, we did not apply any kind of pre-processing such as re-sampling or other normalization. Liver and spleen images were segmented using a U-net-based network architecture successively trained on expert segmentations [17,19]. The PyRadiomics library (version 2.0.1) [20] was used to extract 1288 features each from the liver and spleen, resulting in a total of 2577 imaging features (including a size ratio). These features were classified as size (two-dimensional, three-dimensional, liver to spleen volume ratio), shape (elongation, flatness, sphericity, surface-to-volume ratio), and texture (first order statistics, features derived from various texture descriptor matrices, as well as numerous multi-scale features based on wavelets and Laplacian-of-Gaussian analysis). Note that our feature categories (which are illustrated in Figure 1 with more detailed numbers) explicitly separate size-based features from shape features, although these are often grouped into one category [20]. This is motivated by the fact that liver and spleen sizes are already considered and (at least roughly) reported by radiologists, and shape (such as “nodularity”) is considered a distinct feature by physicians. Hence, we are interested in which of these individual aspects can be covered by our automatic analysis as well.

### 2.5. Statistical Analyses

In describing the clinical characteristics, categorical data (sex, liver etiology, compensation status) were presented as frequency with percentage, while continuous data (age, MELD) were presented as mean with range. In analyzing the radiomic features, we set up experiments for each cohort comparison as follows. For each analysis, 15 repetitions of 5-fold cross-validation were performed, resulting in a total of 75 stratified splits of the imaging data into training and validation sets. For each of these, a Random Forest classifier was trained on the radiomic features of 80% of the imaging data and evaluated on the remaining 20% of patients, obtaining the area under the curves (AUC) as a measure of success. Statistical significance was defined as *p*-value < 0.05 using a random permutation test. In the box plots conveying the results, the boxes indicate the lower and upper quartiles, the solid line indicates the sample median, the dotted line represents the mean, the whisker lines show the upper and lower fences, and dots depict outliers.

## 3. Results

The RPDR queries identified 417 patients for the cirrhosis cohort and 650 patients for the non-cirrhosis IPMN cohort. After fulfillment of study inclusion and exclusion criteria, 90 patients in the cirrhosis cohort and 77 patients in the non-cirrhosis cohort were used for analyses. 

**Figure 2:**
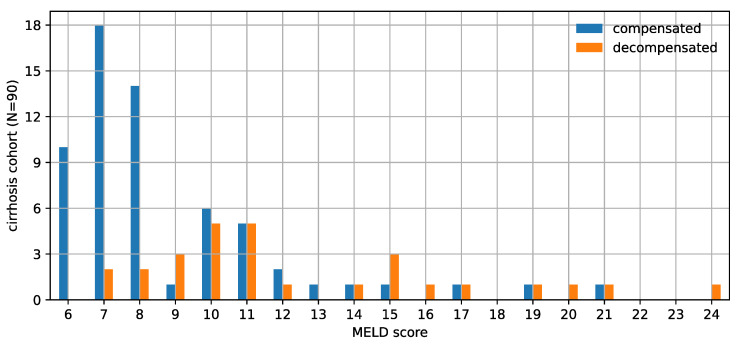
This histogram shows the distribution of the Model for End-Stage Liver Disease (MELD) score and decompensation status of the cirrhosis cohort. This cohort population is biased towards relatively milder liver disease. The imperfect correlation between MELD score and decompensation status is also evident from this chart.

Within the cirrhosis cohort, the mean age was 61.5 years (range 24–83) and 51.1% were male. The etiology of liver disease was 41.1% hepatitis c virus (*n* = 37), 25.6% non-alcoholic steatohepatitis (*n* = 23), 16.7% alcohol (*n* = 15), and 16.7% other (*n* = 15). Median MELD score was 8. At time of scan, 68.9% (*n* = 62) were compensated. Distribution of MELD score and compensation status are shown in Figure 2. Within the non-cirrhosis IPMN cohort, the mean age was 63.5 years (range 36–86) with 16.9% male.

### 3.1. Radiomic Discrimination of Cirrhosis

**Figure 3:**
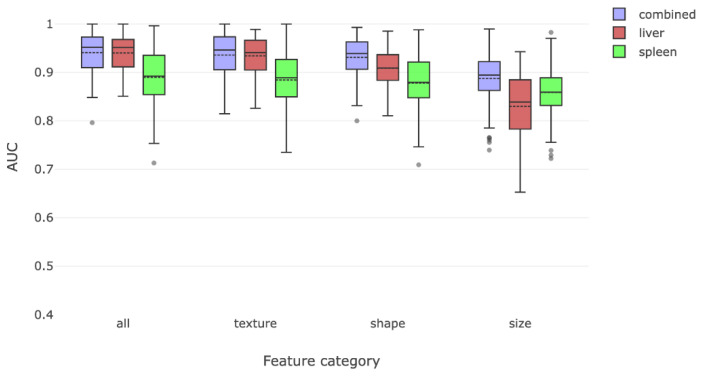
This figure compares the overall radiomic discrimination of cirrhosis between cirrhosis and non-cirrhosis cohorts. Liver features were somewhat more effective in discriminating between the two classes, with texture and shape features being more salient than size features.

The first part of our analyses was to determine whether training of the liver and spleen radiomic features using MR images of patients with and without cirrhosis could enable accurate radiomic recognition of cirrhosis. Training and testing of these liver and spleen radiomic features revealed a combined AUC of 0.94 in identifying cirrhosis. In order to understand the relative importance of liver or spleen derived radiomic features in driving this observation, we re-analyzed the data separately using only liver or only spleen radiomic features. We found that the liver radiomic features were more effective than features in the spleen for discriminating between those with cirrhosis and without cirrhosis (Figure 3).

We then attempted to characterize which of the predefined classes of radiomic features had the best yield for detecting cirrhosis. We separated features into classes measuring shape, texture, and size features. Texture and shape features alone were able to identify cirrhosis with high accuracy. Taken on their own, size features were the least effective in detecting cirrhosis. 

### 3.2. Comparison by Compensation/Decompensation

**Figure 4:**
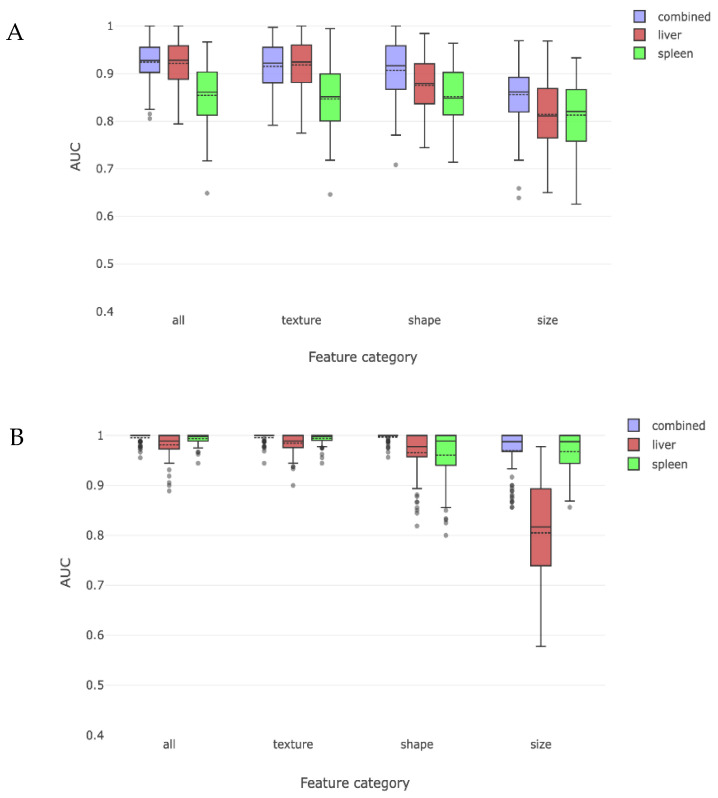
These two figures depict the radiomic discrimination of cirrhosis between non-cirrhosis cohort and either compensated (**A**) or decompensated (**B**) cirrhosis sub-cohorts. Discrimination is more effective for decompensated sub-cohort. Liver features are more effective than spleen features from discriminating compensated subjects, while spleen features become more effective than liver features for decompensated subjects. Texture and shape are again more effective than size features.

We then divided our cirrhosis cohort into compensated and decompensated sub-cohorts and tested whether a combination of liver and spleen radiomic features could lead to correct radiomic detection of cirrhosis when each sub-cohort was compared to the non-cirrhosis cohort. The liver and spleen derived features could distinguish compensated cirrhosis with a combined AUC of 0.92. When analyzed individually, liver derived radiomic features were better at detecting compensated cirrhosis than spleen derived radiomic features (Figure 4a). Similarly, these findings were primarily driven by shape- and texture-based liver radiomic features, with size features being less important. 

This process was repeated for patients with decompensated cirrhosis and compared to those without cirrhosis, yielding an overall AUC of 0.99 for identifying decompensated cirrhosis using combined liver and spleen trained radiomic features. In this case, both liver and spleen features were important when examined separately, with spleen features being as or more important than liver features. When analyzed by type of radiomic feature, all features had similar importance, except for liver derived size features which had an AUC of 0.81 (Figure 4b). 

### 3.3. Comparison by MELD Score

**Figure 5:**
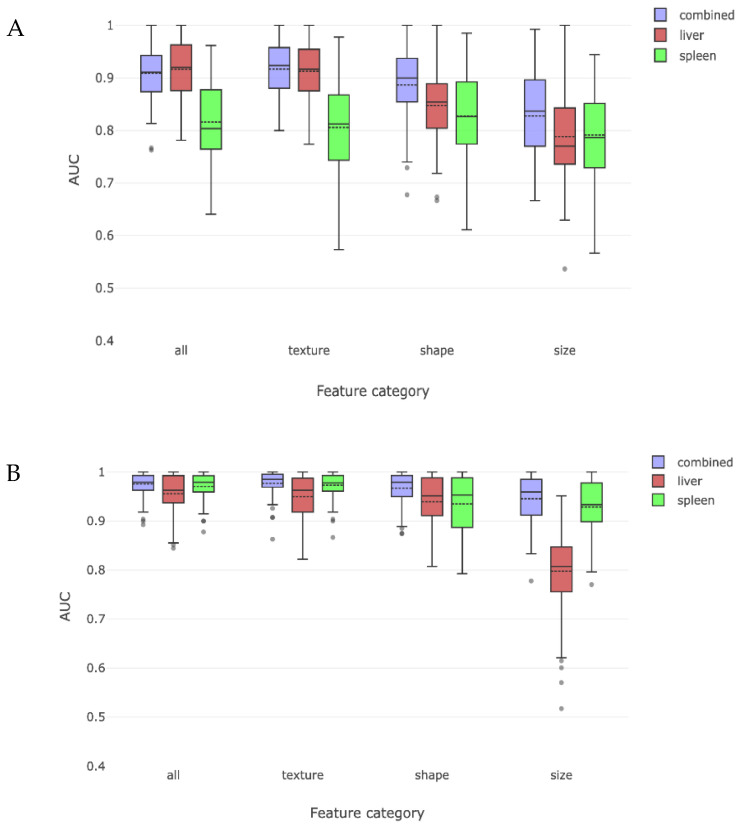
This figure charts radiomic discrimination of cirrhosis between non-cirrhosis cohort and either low MELD (**A**) or high MELD (**B**) cirrhosis sub-cohorts. Results are similar to, but somewhat less effective than, the compensation/decompensation sub-cohort experiments. This difference may be because the MELD score does not in itself predict cirrhosis with absolute accuracy.

We then repeated the process of dividing the cirrhosis cohort into low or high MELD sub-cohorts (stratified at the population median score of 8) and tested the ability of radiomics to detect cirrhosis when each group was compared to the non-cirrhosis cohort. The overall AUC for detecting cirrhosis with a low MELD score was 0.91 and with a high MELD score was 0.98. The effect of liver and spleen derived features alone and the impact on our predefined radiomic features are shown in Figure 5. When low MELD results are compared to the previously obtained compensated results (Figure 4), it was not as strong in detecting cirrhosis. Similar observations were made for high MELD and decompensated results. 

## 4. Discussion

We observed that liver and spleen radiomic features had a high AUC of 0.94 in identifying patients with cirrhosis when MR imaging scans were compared to those without cirrhosis. This is an improvement from current approaches in reading MR imaging [1,2] and this result is consistent with another study that used only liver radiomic features in a younger population [15]. We found that this discrimination was primarily driven by texture and shape based radiomic features with size-based features being less important. The reliance on texture and shape based radiomic features is probably reflective of structural changes that may occur with cirrhosis and portal hypertension such as surface nodularity, caudate lobe enlargement, posterior notch, and splenomegaly [2,21,22,23,24]. 

To better understand how liver disease severity may affect the yield of detecting cirrhosis using radiomics when compared to those without cirrhosis, we stratified the patients with cirrhosis by clinical compensation/decompensation and MELD score, since these metrics are commonly used markers of cirrhosis severity. When using clinical compensation/decompensation as a proxy for cirrhosis severity, detection of cirrhosis remained high with AUC 0.92 for those with compensated cirrhosis and was mainly driven by liver derived features rather than spleen derived features with radiomic shape and texture features performing better than size-based features. These findings are consistent with compensated liver disease being reflective of structural liver changes. Interestingly, the spleen features had a non-trivial AUC of 0.85 suggesting that there may be structural changes in the spleen from portal hypertension which can occur in compensated cirrhosis. As we could not assess portal pressures in this retrospective study, it is difficult to say whether these findings in our compensated cirrhosis cohort are reflective of those with mild or clinically significant portal hypertension. Among those with decompensated cirrhosis, liver and spleen derived radiomic features had an AUC of 0.99 for capturing cirrhosis. Notably, spleen features were as effective or more effective than the liver features in their predictive power. Decompensation is associated with worsening portal hypertension and resultant hypersplenism. However, in all cases, radiomic based shape and texture features were more important than size-based features. Change in spleen size does not sufficiently account for the importance of the spleen features in predicting severe cirrhosis. While spleen elastography has been utilized to assess portal hypertension [25], we are unaware of other studies that have associated quantitative changes in spleen texture and shape with severe cirrhosis. Given that radiological assessment of cirrhosis typically reports only spleen size as a single gross quantitative metric, further research into physiological changes in the spleen due to the progression of cirrhosis seems warranted.

The experiments that used radiomics to distinguish between the non-cirrhosis subject cohort and low- and high-MELD sub-cohorts had comparable results to the experiments that used clinical compensation/decompensation, although the radiomics technique did not perform as well in the MELD-based experiments. This reduced performance may in fact be a strength and not a shortcoming. Previous research suggests that MELD has an AUC of approximately 0.8 for liver related mortality; MELD’s ability to accurately predict cirrhosis is good but not without limitations [26]. Our experimental results suggest that radiomics derived liver and spleen features might capture complementary or even more accurate information about liver disease severity than is possible with MELD. A future combination of image-derived quantitative biomarkers and established measures such as MELD score has the greatest potential to provide a more accurate clinical estimate of liver disease severity. 

Strengths of this study are that radiomic analyses were performed on standardized MR images in patients with and without cirrhosis by using the same type of MR scanner, protocol, contrast regimen and by analyzing the same post-contrast image. We also excluded scans that may affect interpretation of liver features such as hepatocellular carcinoma and prior hepatic resection or ablation. An advantage of our study was that it not only tested the ability of trained liver and spleen radiomic features to discriminate cirrhosis but also how that was affected by decompensation status and MELD score, which are commonly used (albeit far from perfect) proxies for liver disease severity. Importantly, radiomics worked well in identifying cirrhosis in our study population of early-stage cirrhosis as most patients were compensated with median MELD 8. We also classified liver and spleen radiomic features by size, shape, and texture in order to better understand the predominant features in radiomic detection of cirrhosis. 

Limitations of this study are that it is a single-center retrospective study with a small sample size, raising the possibility of selection bias in training and testing of radiomic features. While we attempted to organize and describe liver and spleen radiomic features in simplified terms of size, shape, and texture for ease of understanding radiomics, this combination and characterization of radiomic features may be an oversimplification. By masking out all parts of the image data except liver and spleen, our analysis does not include other radiographic findings seen in the rest of the abdomen. Although we were able to divide patients into compensated and decompensated cohorts, we could not retrospectively divide the compensated cirrhosis cohort into substages of mild portal hypertension or clinically significant portal hypertension which should be characterized further in future radiomic studies. Additionally, it is possible that some patients within the non-cirrhosis cohort may have had undiagnosed liver disease despite our effort to screen out these patients. Furthermore, as our findings have not been tested in MR images acquired from different MR scanners and contrast protocols, the generalizability of our method to a more heterogeneous collection of scan data is unknown. For example, it is possible that radiomic texture analysis may be sensitive to scanner and protocol differences. On the other hand, shape features performed well in all cases and are likely relatively stable to scanner and protocol changes. Further study in this area is warranted.

In summary, our study demonstrates the strong ability of trained MR liver and spleen radiomic features for detecting cirrhosis even after stratification by MELD or clinical compensation/decompensation. The prospect of using radiomics to enhance existing MR images has the potential to improve recognition of cirrhosis on commonly used imaging studies. Our finding that radiomic discrimination of cirrhosis primarily relied on shape and texture of the liver and spleen suggests that radiomics may detect subtle physiologic activity of cirrhosis and portal hypertension which could be used to provide objective radiographic measures of cirrhosis severity. The objective nature of our method makes it potentially suitable as a tool to compare liver severity across patients for purposes including organ allocation and transplant prioritization. While this study demonstrates the predictive ability of radiomics to detect cirrhosis, our method provides the basis for future developments in areas including continuous metrics of liver severity and methods to cluster similar patients. More research is warranted for using radiomics as a quantitative biomarker in diagnosing cirrhosis and complementing existing markers of cirrhosis severity.

## Data Availability

The data presented in this study are available on request to the corresponding author.
